# GLUCOCORTICOID-INDUCED BONE DISEASE: MECHANISMS AND IMPORTANCE IN PEDIATRIC PRACTICE

**DOI:** 10.1590/1984-0462;2017/;35;2;00007

**Published:** 2017-05-29

**Authors:** Emanuel Sávio Cavalcanti Sarinho, Verônica Maria Pinho Pessoa Melo

**Affiliations:** a Universidade Federal de Pernambuco (UFPE), Recife, PE, Brasil.

**Keywords:** glucocorticoids, osteoporosis, fractures, children, adolescents

## Abstract

**Objective:**

To describe mechanisms by which glucocorticoids cause osteoporosis, with fracture risk, combining this learning with a possible professional behavior change.

**Data sources:**

A systematic search on SciELO, PubMed, Scopus, and Medline databases was carried out for consensus, review articles, including systematic reviews and meta-analysis, which were published in English, between 2000 and 2016. Keywords used on the search were the following: glucocorticoids, fractures, osteoporosis, bone health, vitamin D, children, and adolescents.

**Data synthesis:**

The review was divided into four topics: 1) introduction, with a brief focus on pediatric fractures; 2) osteoporosis in children and adolescents, highlighting it as a silent cause of fractures; 3) glucocorticoids and secondary bone disease, describing deleterious mechanisms of this steroids group on bone structure; 4) molecular effects of glucocorticoids excess on bone, with details about the harmful mechanisms on bone molecular level.

**Conclusions:**

Glucocorticoids excess determines early bone disease, favoring the occurrence of fractures. Thus, a child or an adolescent who uses glucocorticoids, especially systemically and chronically, but also repeats cycles at high cumulative doses of the medication, needs care and guidance related to bone health at the onset of treatment. On the other hand, the presence of fractures, even if related to trauma, can be a sign of underlying and unknown bone fragility, which may be secondary to the use of glucocorticoids and/or vitamin D deficiency.

## RESUMO

**Objetivo:**

Descrever os mecanismos pelos quais os glicocorticoides provocam osteoporose, com risco consequente de fraturas, integrando esse conhecimento a uma possível mudança de conduta dos profissionais de saúde.

**Fontes de dados:**

Foi realizada pesquisa aprofundada nas bases de dados (SciELO, PubMed, Medline e Scopus), buscando consensos, artigos de revisão, incluindo revisões sistemáticas e meta-análises, publicados em inglês, entre 2000 e 2016. As palavras-chaves utilizadas na busca foram glicocorticoides, esteroides, fraturas, osteoporose, saúde óssea, crianças e adolescentes.

**Síntese dos dados:**

A revisão foi dividida em quatro tópicos principais: 1) introdução, com breve enfoque nas fraturas em pediatria; 2) osteoporose em crianças e adolescentes, destacando-a como causa silenciosa de fraturas; 3) glicocorticoides e doença óssea secundária, com a descrição dos mecanismos deletérios desse grupo de esteroides na estrutura óssea; 4) efeitos moleculares do excesso de glicocorticoides no osso, com o detalhamento dos mecanismos nocivos a nível molecular do tecido ósseo.

**Conclusões:**

Os glicocorticoides em excesso determinam doença óssea precoce, favorecendo a ocorrência de fraturas. Dessa forma, uma criança ou adolescente que requer corticoterapia, sobretudo crônica e sistêmica, mas também em ciclos repetidos com doses cumulativas altas, necessita de cuidados e orientações relacionados à saúde óssea logo ao início do tratamento. Por outro lado, aqueles com fratura, mesmo entrelaçada a um trauma, podem sinalizar fragilidade óssea subjacente e desconhecida, incluindo a secundária ao uso de glicocorticoides e à deficiência de vitamina D.

## INTRODUCTION

European studies show that 30-50% of young people will suffer at least one fracture up to 17 years of age, with an annual incidence of approximately 103-257 per 10,000 individuals. Fractures occur most frequently among boys (61-64%) in the age range of 13-14 years. Among girls, the predominant age range is 11-12 years. The bones of the forearm are the most frequently affected.^1^ ^,^ ^2^ ^,^ ^3^ ^,^ ^4^ ^,^ ^5^ Fractures occurred in 12.1% of accidents involving Brazilian children aged 0-9 years, mostly caused by falls at home.^6^

In addition to the intensity of the trauma, a variety of intrinsic and extrinsic factors - including genetics, puberty, adequate intake of calcium, protein, and vitamin D, obesity, physical activity, chronic inflammatory morbid conditions, and use of medications - affect the occurrence of a fracture. Genetic inheritance, which accounts for 60-80% of bone mass, is the most important factor.^7^

## OSTEOPOROSIS IN CHILDREN AND ADOLESCENTS

A fracture may indicate underlying bone fragility, that is, osteoporosis, which is not restricted to the elderly. The National Institute of Health (NIH), an American organization, established the current definition of osteoporosis in 2001 after a consensus: “a skeletal disorder characterized by compromised bone strength predisposing to an increased risk of fracture.”^8^ Osteoporosis is silent, prevalent worldwide, and its morbidity and mortality are associated with the fractures it causes.^9^

Bone, which is apparently inert, has protective and supportive functions, and actively participates in the mineral metabolism. Therefore, maintaining bone health is essential as the bone communicates with the immune system, and it is considered an endocrine organ, since it produces hormones such as fibroblast growth factor 23 (FGF23) and osteocalcin.^10^ ^,^ ^11^

Two types of bones are identified in human beings: cortical bone, also known as compact bone, which represents 85% of the skeleton, and has protective and mechanical function; and trabecular, also known as spongy bone, which provides strength and mostly has metabolic functions.^12^ ^,^ ^13^ Bone tissue is composed of a matrix (mainly collagen), minerals (especially calcium, phosphorus, and magnesium), and cells (osteoblasts, osteocytes, and osteoclasts). Bone mineral is accrued throughout childhood and adolescence, and reaches a plateau at around the end of the second decade, which is named peak bone mass (PBM) and will determine bone mass throughout life.^14^

It is estimated that 10% increase in PMO can delay senile osteoporosis in 13 years.^15^ Approximately 40-60% of adult bone mass is accrued in adolescence, and 25% of the PBM is accrued during the period of two years around the growth spurt.^15^ ^,^ ^16^ Even without increased bone loss, an adult can develop osteoporosis because he or she has not reached their PBM in childhood and adolescence.^17^ As mentioned earlier, the genetic inheritance accounts for 75% of the variance in the PBM.^7^ ^,^ ^18^

Bone mineral density is the amount of mineral mass per area or bone volume.^18^ However, the concept of bone health is more comprehensive and is not only related to bone mass. The latter is considered the most important and more easily measurable parameter.^17^ Morphology (size and proportion of cortical and trabecular compartments) and quality (microarchitecture, turnover, and mineralization) also contribute to the bone strength.^19^

Three major techniques are used to assess bone mineral density: dual-energy X-ray absorptiometry (DXA), quantitative computed tomography (QCT), and quantitative ultrasound.^20^ DXA, whose results are expressed in grams/cm^2^, uses parallel beam radiation that pass through the soft tissue and bone and are captured by a sensor located oppositely.^21^ Therefore, it depicts the bone in two-dimensional form. Despite being the most used technique, DXA does not assess bone geometry, does not distinguish trabecular bone from cortical bone, and in pediatrics, it requires comparison with healthy individuals of the same age, sex, and ethnicity, analyzed by similar equipment and software.^22^

Peripheral quantitative computed tomography (pQCT) and high-resolution peripheral quantitative computed tomography (HR-pQCT), which are techniques restricted to research using low radiation, assess the bone in three-dimensional form, quantify the contribution of geometry and mass to bone strength, and differentiate the trabecular bone from the cortical bone.^22^ ^,^ ^23^

Quantitative ultrasound is a quick, inexpensive, and simple technique. It provides information on the elasticity and bone structure, but the quality of the results, which are dependent on the operator, still does not allow to replace DXA.^20^ However, training and the development of professionals can be valuable considering the advantages of this examination.

Osteoporosis has diagnostic peculiarities among children and adolescents. Biochemical markers of bone turnover may be affected by many factors, and there are difficulties in the interpretation of densitometry related to growth and puberty, preventing the isolated use of this tool for diagnostic definition.^20^ ^,^ ^22^ Therefore, experts gathered in a worldwide consensus established the diagnosis of osteoporosis in children, in addition to the indications, interpretation, and the use of bone densitometry in this age group.^22^ ^,^ ^24^ The isolated finding of one or more vertebral compression fractures (in the absence of local disease or severe trauma) or a Z score (adjusted for age and gender) of bone mineral density at or below -2, necessarily associated with a clinically significant history of fracture (two or more long bone fractures up to 10 years of age, or three or more fractures of long bones below 19 years of age), are diagnosis of osteoporosis in this age group.^22^ ^,^ ^25^

The actual incidence and prevalence of osteoporosis in the pediatric population are not well defined; however, it is known that osteoporosis may compromise both sexes and occur at any age, being classified as primary or genetic (whose main representative is osteogenesis imperfecta) and secondary to conditions generally associated with each other (chronic inflammation, neoplasms, prolonged immobilization, use of certain medications, hormonal and nutritional disorders).^10^ ^,^ ^26^ Anticonvulsants, anticancer (especially methotrexate), calcineurin inhibitors, anticoagulants, and glucocorticoids (GC) are drugs associated with the development of osteoporosis,^10^ with emphasis to the last ones, which are widely used in pediatric prescription.

## GLUCOCORTICOIDS AND SECONDARY BONE DISEASE

GC are steroid hormones physiologically produced by the adrenal glands and synthesized for intermittent or continuous systemic and topical use. GC are recognized by the significant anti-inflammatory and immunomodulatory activities, being used to control the pathogenesis of autoimmunity and/or inflammation in a variety of diseases. They have been increasingly used in the pediatric age group.^27^ A study carried out in the UK found that 1.2% of children received at least one prescription of oral corticosteroids within one year, mostly for treating asthma attacks.^28^

This is the group most assisted by the general pediatrician. Asthma is a chronic, obstructive, and inflammatory pulmonary disease, with differences in clinical presentation, severity, and response to treatment, which is usually based on the use of GC.^29^ The Global Initiative for Asthma (GINA), an organization that brings together pulmonologists, and annually prepares guidelines for asthma management, has recommended therapy in stages or “steps” for the control of asthma, whose severity classification (mild, moderate, and severe), is established on the basis of the response to treatment, and may change over time.^30^ The initial treatment for most patients with asthma (except mild cases in preschool children) includes inhaled GC for several months and with the lowest effective daily dose. On the other hand, oral systemic GC is part of rescue therapy in asthma attacks (cycles of 1 and 2 mg/kg/day of prednisolone for three to five days) and continuous treatment of the most severe cases.^30^

Chronic inflammatory diseases of the connective tissue, kidneys, nervous system, and digestive tract, in addition to cancer and transplants, also require therapy with oral systemic GC, but continuously and for more than three months. Juvenile idiopathic arthritis, systemic lupus erythematosus, juvenile dermatomyositis, Crohn’s disease, and nephrotic syndrome are the main examples.^31^ ^,^ ^32^ ^,^ ^33^ The chronic inflammatory disease itself promotes the release of cytokines, malnutrition, prolonged immobilization, loss of muscle mass, delayed puberty, and reduced sun exposure, affecting negatively bone health.^31^ ^,^ ^33^ However, clinical trials on volunteers showed that there is a direct corticoid action in decreased bone formation, independent of inflammation.^34^ In any case, the sum of the harmful actions of the disease and steroid therapy converges to compromise bone health.

If used chronically, GC are considered the main cause of secondary and iatrogenic osteoporosis.^27^ ^,^ ^35^ However, the potential for fractures is often disregarded by the professional who prescribes the GC and ignored by the patient and the family. The relative risk increases with dose and duration of steroid therapy, whereas the absolute risk is determined by a variety of associated clinical conditions. However, low daily doses of 2.5 mg of prednisolone, in intermittent and repeated cycles, may have cumulative and also harmful effect.^36^ The risk is higher in the first three months of continuous therapy and slowly decreases after its conclusion; however, it does not seem to return to normality.^37^ In addition, this rapid increase in the risk of fracture is not registered by densitometry tests, which suggests a more significant change in bone quality over quantity.^35^ Therefore, the absence of alterations in bone densitometry does not rule out the existence of GC-induced osteoporosis.

Extensive study of a British cohort, including individuals aged 4-17 years, revealed that the annual use of more than four cycles of oral systemic GC increased fracture risk, whereas another cohort study, with the same age range and in the same country, revealed an association of increased risk of fracture with severity of asthma, and not with the use of inhaled steroid.^28^ ^,^ ^38^

GC-induced bone disease mainly affects trabecular bone, justifying the higher occurrence of vertebral and rib fractures.^40^ However, a British study revealed that the humerus was the most fractured bone among children who used four or more short cycles of oral corticosteroids.^28^ Experimental, molecular, and genetic research has been elucidating the mechanisms of action of GC excess on bones. These steroids have a catabolic effect on the muscle and the vitamin D, which compromises bone mineralization.^41^ ^,^ ^42^ Bone and muscle form a unit, with mutual trophic influence. Moreover, chronic corticosteroid therapy promotes adipogenesis and obesity, with capture and reduction of vitamin D.^43^ ^,^ ^44^

**Figure 1:**
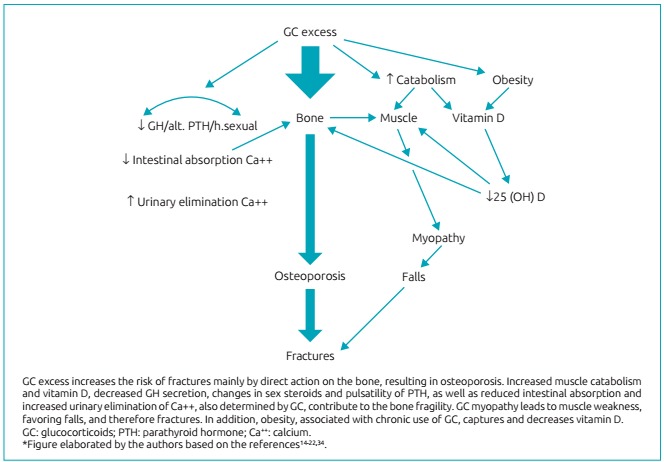
Pathogenesis of fractures related to glucocorticoids (GC) excess.

Decreased intestinal calcium absorption, increased urinary calcium elimination, reduction in the secretion of growth hormone (GH), and changes in the metabolism of sex steroids and in parathyroid hormone (PTH) pulsatility are other indirect negative effects of GC on bone health.^40^ However, the bone tissue itself is a target for the GC. Research has shown that the direct action of these steroids on bone cells is the main mechanism in the genesis of the resulting osteoporosis.^37^ ^,^ ^40^ Figure 1 summarizes the pathogenesis of secondary fracture to GC excess.

## MOLECULAR EFFECTS OF GLUCOCORTICOIDS EXCESS ON BONE

GC’s effects occur by four mechanisms: the classic genomic (the most important), which involves the cytosolic GC receptors (cGCR), and is divided into two processes, transrepression and transactivation; nongenomic secondary, which is also initiated with cGCR; nongenomic executed by membrane receptors (mCGR); and nonspecific nongenomic, resulting from interactions with cell membranes (including the organelles).^45^ ^,^ ^46^ ^,^ ^47^ The anti-inflammatory and immunomodulatory actions are derived from these processes, as well as adverse effects, which are determined by one or more of the four mechanisms.

The polymorphism of the genes related to these receptors may contribute to differences in the intensity of bone diseases and fractures induced by the use of GC.^47^ Clinical observation and studies on cell metabolism of GC also identified several sensitivity to these hormones, related to intracellular enzyme system 11β-hydroxysteroid dehydrogenase (11β-HSD), specifically to type 1 (11β-HSD1), which converts inactive forms of corticosteroids (cortisone and prednisone) in active forms (cortisol and prednisolone).^27^ ^,^ ^48^ Immunohistochemical and *in situ* hybridization studies showed 11β-HSD1 expression in osteoblasts increasing with age, which favors the greater concentration of GC in these cells, and which may undergo modulation by cytokines, growth factors, and other enzymes.^36^

Increased expression of 11β-HSD1 enzyme is considered a risk factor for GC-induced osteoporosis.^12^ In its active form, the GC binds with a receiver (GRα or GRβ), a member of the nuclear receptor super family,^49^ and migrates from the cytoplasm to the nucleus, where it can bind to glucocorticoid response elements (GREs), and to other transcription factors (activator protein 1 [AP1], nuclear factor κB [NF-kB], signal transducer, and activator of transcription 5 [STAT5]), resulting in transactivation or transrepression.^36^ ^,^ ^49^ Therefore, the actions of GC at the genome level, which modifies gene expression, will impact the structure of bone tissue.

**Figure 2:**
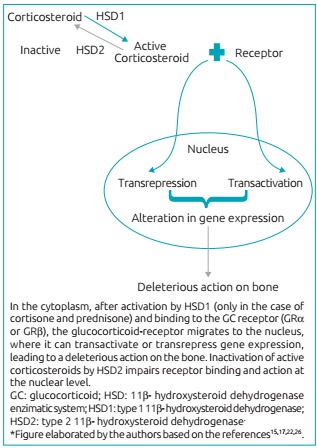
Genomic mechanism of action of GC and the hydroxysteroid dehydrogenase (HSD) enzymatic system.

The other side of the HSD system equation is composed of GC-inactivating enzyme and HSD 2. The sensitivity of different types of GC to this enzyme varies, and dexamethasone, by having a fluorine atom at the 9α position of the B ring, with site of HSD2 blocked, is the steroid which is most resistant to such inactivation, and therefore is what causes osteoporosis the most.^35^ Figure 2 outlines the 11β-HSD enzyme system and the connection with the genomic mechanism of GC action. The result of activation or the resistance to inactivation of GC excess in bone contributes to osteoporosis.

Owing to this nuclear action, GC interferes in the formation, differentiation, destruction, and survival of bone cells. They perform a metabolic control by modulation of various regulatory factors and of matrix proteins, such as collagen, alkaline phosphatase, the receptor activator of nuclear factor kappa B - NF-kB (RANK), the receptor activator of nuclear factor kappa B ligand (RANKL)/osteoprotegerin (OPG), osteocalcin, osteopontin (OPN), the pro- and antiapoptotic proteins, in addition to growth factors and cytokines, which interfere significantly in bone metabolism.^49^

Experimental studies have revealed that an important bone signaling pathway, the Wnt/β-catenin pathway, is impaired by excess GC. The term Wnt derives from the merge of the name of 2 *loci* involved, int-1 and Wg, but also comprises other protein signaling pathways, being the Wnt/β-catenin which is the main signaling pathway.^50^ The Wnt binds to a double set of receivers (proteins 5 and 6 related to the low density lipoprotein receptor [Lrp5 and Lrp6]), and to a family member of the frizzled proteins, activating dishevelled protein, and receiving the binding of Axin protein, which binds to the proteins of degradation complex (especially glycogen synthase kinase-3b [GSK-3b]), preventing the phosphorylation and inactivation of β-catenin, which accumulates within the cell.^50^ ^,^ ^51^

The β-catenin accumulated and agglomerated in the cell cytoplasm translocates into the nucleus, where it binds to members of the family of transcription factors (T-cell factor/lymphocyte elongation factor [TCF/Lef]), regulating gene expression to favor bone homeostasis through the targeting of differentiation of mesenchymal stem cells into osteoblasts and inhibiting apoptosis of osteoblasts and osteocytes.^50^ ^,^ ^51^ In addition, by promoting the expression of osteoprotegerin (OPG), competitor of RANKL, inhibits osteoclastogenesis.^51^ Therefore, this signaling pathway contributes to the accumulation of bone mass. Bone response to mechanical overload is also influenced by Wnt β-catenin.^50^

An experimental study found that dexamethasone inhibits all Wnt β-catenin pathway from the cell surface to nuclear translocation.^52^ In addition, experiments with animals showed that the use of GC, especially for prolonged periods, increases the expression of antagonists of Wnt β-catenin pathway (sclerostin and protein 1 of protein Dickkopf family [DKK1]), mainly produced by osteocytes.^51^ These findings reveal the complexity of the negative actions of these steroids in this protein pathway, which partly constitutes its harmful effects on the bone.

**Figure 3:**
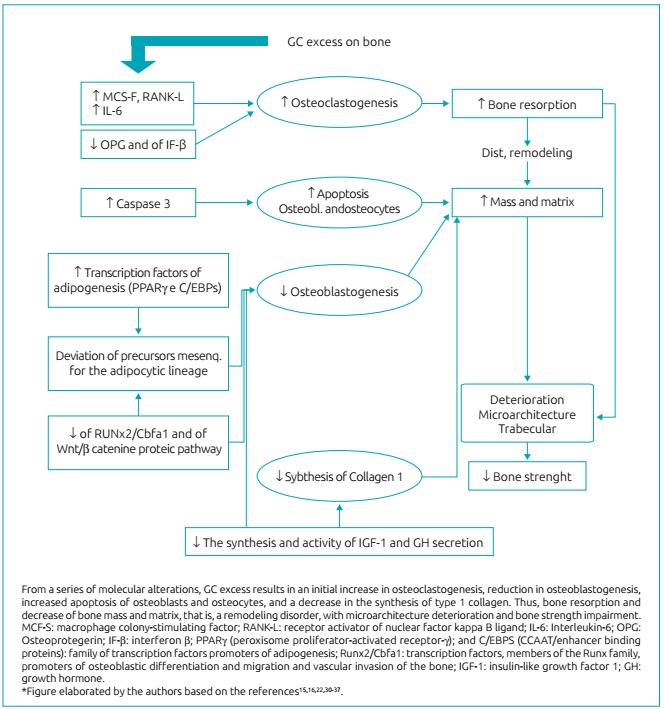
Synthesis of the main deleterious actions of glucocorticoids excess in the bone.

Figure 3 illustrates the main actions of GC excess on bone cells. The increased expression of cytokines that promotes osteoclastogenesis, such as macrophage colony-stimulating factor (M-CSF) and RANKL, associated with decreased OPG (which has an opposite effect) are responsible for the initial rapid increase in bone resorption. This phase is followed by a slower and enduring decrease in bone formation, which is secondary to the decrease of osteoblastogenesis, to the increase in apoptosis of osteoblasts, and to the suppression of type I collagen production (main component of the bone matrix) and stimulating factors formation (such as the IGF-1).^31^ ^,^ ^37^ ^,^ ^47^ Therefore, the initial phase of resorption dominance is followed by the chronic phase of compromising the formation.

GC excess causes, in brief, decreased osteoblastogenesis, increased apoptosis of osteoblasts and osteocytes, and temporary increase of osteoclastogenesis and osteoclast survival.^37^ The favoring of apoptosis of osteocytes, which are sensors for damage and repair support, interferes in the bone remodeling, with reduction in the replacement of the bone excavated by osteoclasts.^27^ ^,^ ^31^ ^,^ ^47^ ^,^ ^53^ ^,^ ^54^ A fact that stands out is the early impairment of bone strength in disproportion to the intensity of decreased bone mass, measured by densitometry tests.

Experimental studies show that OPG (decreased by GC) promotes endothelial proliferation (angiogenesis), whereas the RANKL oppositely inhibits angiogenesis.^55^ In addition, the osteoblasts and osteocytes produce vascular endothelial growth factor (VEGF) and the increase in its apoptosis, secondary to GC excess, would be an additional factor to promote the breakdown of bone vasculature (connected to the canalicular fluid flow), with a loss of hydraulic support and decreased bone strength in relation to the bone mass.^56^ The GC excess seems to affect the bone in many different ways, in a sequence of damages, related to dose and duration of exposure, but also dependent on the individual genetic characteristics.

## CONCLUSIONS

The deepening of research techniques has enabled valuable findings for understanding the action of GC, especially if chronically used, at cellular bone level. This opens a pathway for more research concerning therapy and prevention for the resulting bone disease. The professional who prescribes steroid and those who assist children and adolescents with fractures should be aware of the possibility of associated bone fragility, to direct their research and improve their preventive and therapeutic approach.

## RECOMMENDATIONS

A child or adolescent requiring chronic and systemic corticosteroid therapy in repeated cycles, with high cumulative doses (> 1 g/year), needs care and guidance related to bone health, as soon as the therapy is initiated. The correct administration, including the time, duration, and mode of discontinuing treatment, should be clarified.

Nutritional follow-up is essential to prevent obesity, but also to ensure adequate intake of calcium (1,300 mg/day between 9 and 18 years) and proteins, as well as to maintain sufficient levels of vitamin D, with a minimum daily intake of 600 IU, after the first year of life.^22^ The recommendation for the daily exposure to sunlight for approximately 20 minutes, must be accompanied by serum levels of 25(OH) of vitamin D, at the onset of therapy, and every three to six months, depending on the results. If serum levels are below 30 ng/mL, the oral replacement with medication is recommended.

The regular and supervised physical activity, although it may lead to accidents,^57^ should be recommended, considering the beneficial action on bone and muscle strength, in addition to the prevention of obesity.

The suspicion of spontaneous fractures, especially invertebrae (evidenced by pain or loss of height), must be latent and requires radiological investigation.^22^ The peculiarity of vertebral involvement highlights the importance of clinical monitoring and routine anthropometry. The professional who cares for a child or adolescent with fracture, especially after a mild trauma, should be alert to the possibility of underlying bone fragility, which includes questioning the use of GC and investigating deficiency of vitamin D.

The physician should not fear to prescribe corticosteroids for asthma and other inflammatory diseases if well indicated. He or she should only be aware that the need must be precise and that the use of repeated cycles, with cumulative doses greater than 1 g/year, may interfere with bone health. The search for synthetic forms of GC, that act only on the anti-inflammatory and immunomodulatory mechanisms, without the harmful side effects, is a present hope.^49^ ^,^ ^58^
